# Supplementary material for: Efficient rational modification of non-ribosomal peptides by adenylation domain substitution
Source: Nat Commun. 2020 Sep 11;11:4554. doi: 10.1038/s41467-020-18365-0 (PMC7486941; doi:10.1038/s41467-020-18365-0)
Supplement: Supplementary file 1 — Supplementary Information [file 41467_2020_18365_MOESM1_ESM.pdf]

Calcott et al. Efficient rational modification of non-ribosomal peptides by adenylation domain substitution

Supplementary Information:

Supplementary Figures 1-14

Supplementary Tables 1-3

Supplementary References

Correspondence to david.ackerley@vuw.ac.nz

Supplementary Figure 1. Comparison of the C domains from modules Pa8 and Pa11. Related to Figure 2a. (a) Amino acid sequence alignment of the C domain from Pa11, the second module of PvdD, and the C domain from Pa8, the first module of PvdJ. The alignment is delineated into three regions defined by low homology stretches (highlighted in red, blue or green shading, respectively, consistent with the variable residue colouring scheme used in Fig. 2a). Conserved motifs are underlined in bold. (b) Homology model of the C domain from Pa11 identifying in colour the low-homology regions identified in panel a, and with the catalytic histidine residue shown in cyan. (c) A zoomed image of the active site of the homology model.

a

```
Pa11_Thr    QQPLALSFAQERQWFLWQLEPESAAYHIPSALRLRGRLDVDALQRSFDSLVARHETLRTR     60
Pa8_Lys     MDNLPLSYAQERQWFLWQLEPESSAYHIPTALRLRGRLDIASLQRSFAALVERHESLRTR     60
             : * **:***************:*****:*********: :***** :** ***:****

Pa11_Thr    FRLEGGRSYQQVQPAVSVSIER---EQFGEEGLIERIQAIVVQPFDLERGPLLRVNLLQL     117
Pa8_Lys     IARMGDEWVQVVSADVSLALEVEVQRGLDEQRLLERVEAEIARPFDLEQGPLLRVTLLEV     120
            :   *..  * *.  **:::*    . :.*: *:**::* :.:*****:******.**::     Region 1

Pa11_Thr    AEDDHVLVLVQHHIVSDGWSMQVMVEELVQLYAAYSQGLDVVLPALPIQYADYALWQRSW     177
Pa8_Lys     DADEHVLVMVQHHIVSDGWSMQLMVEELVQLYAAYSQGLDVVLPALPIQYADYALWQRSW     180
              *:****:*************:*************************************

Pa11_Thr    MEAGEKERQLAYWTGLLGGEQPVLELPFDRPRPARQSHRGAQLGFELSRELVEAVRALAQ     237
Pa8_Lys     MEAGEKERQLAYWTGLLGGEQPVIELPLDHPRQPLRSYRGAQLDLELEPHLALALKQLVQ     240
            ***********************:***:*:**   :*:*****.:**. .*. *:: *.*
                                                                             Region 2
Pa11_Thr    REGASSFMLLLASFQALLYRYSGQADIRVGVPIANRNRVETERLIGFFVNTQVLKADLDG     297
Pa8_Lys     RKGVTMFMLLLASFQALLHRYSGQADIRVGVPIANRNRVETERLIGFFVNTQVLKADING     300
            *:*.: ************:**************************************::*

Pa11_Thr    RMGFDELLAQARQRALEAQAHQDLPFEQLVEALQPERNASHNPLFQVLFNHQSEIRSVTP     357
Pa8_Lys     RMGFDELLAQARQRALEAQAHQDLPFEQLVEALQPERSLGHNPLFQVMFNHQADSRSANQ     360
            *************************************. .*******:****:: **..

Pa11_Thr    EVQLEDLRLEGLAWDGQTAQFDLTLDIQEDENGIWASFDYATDLFDASTVERLAGHWRNL     417
Pa8_Lys     GVQLPGLSLERMEWRSSSVAFDLTLDVHEAEDGIWASFGYATDLFEASTVERLARHWQNL     420
             *** .* ** : * ..:. ******::* *:******.******:******** **:**     Region 3

Pa11_Thr    LRGIVANPRQRLGEL      432
Pa8_Lys     LRGIVAEPGRPVAEL      435
            ******:* : :.**
```

b c

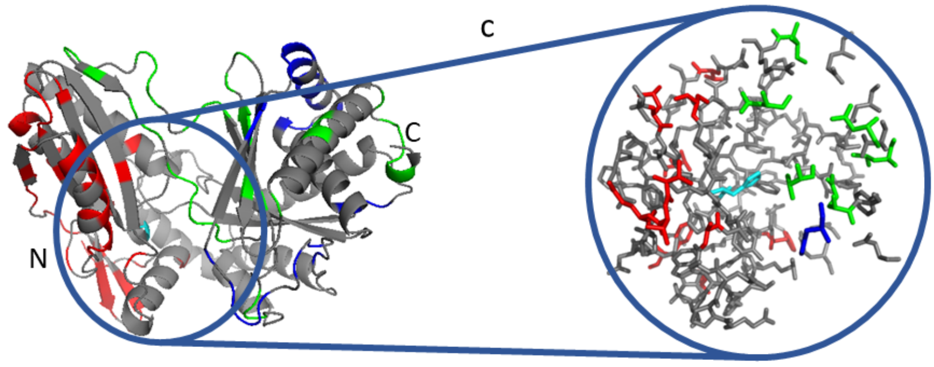

Supplementary Figure 2. Amino acid alignment highlighting the differences between shuffled C domains and the original Pa11-Thr C domain. Related to Figure 2c. The C domains are labelled with either a T or K for each variable region to indicate whether the region was sourced from the Pa11-Thr C domain (TTT) or Pa8-Lys C domain (KKK), respectively. Image created using Geneious version 8.1 (Biomatters. Available from http://www.geneious.com).

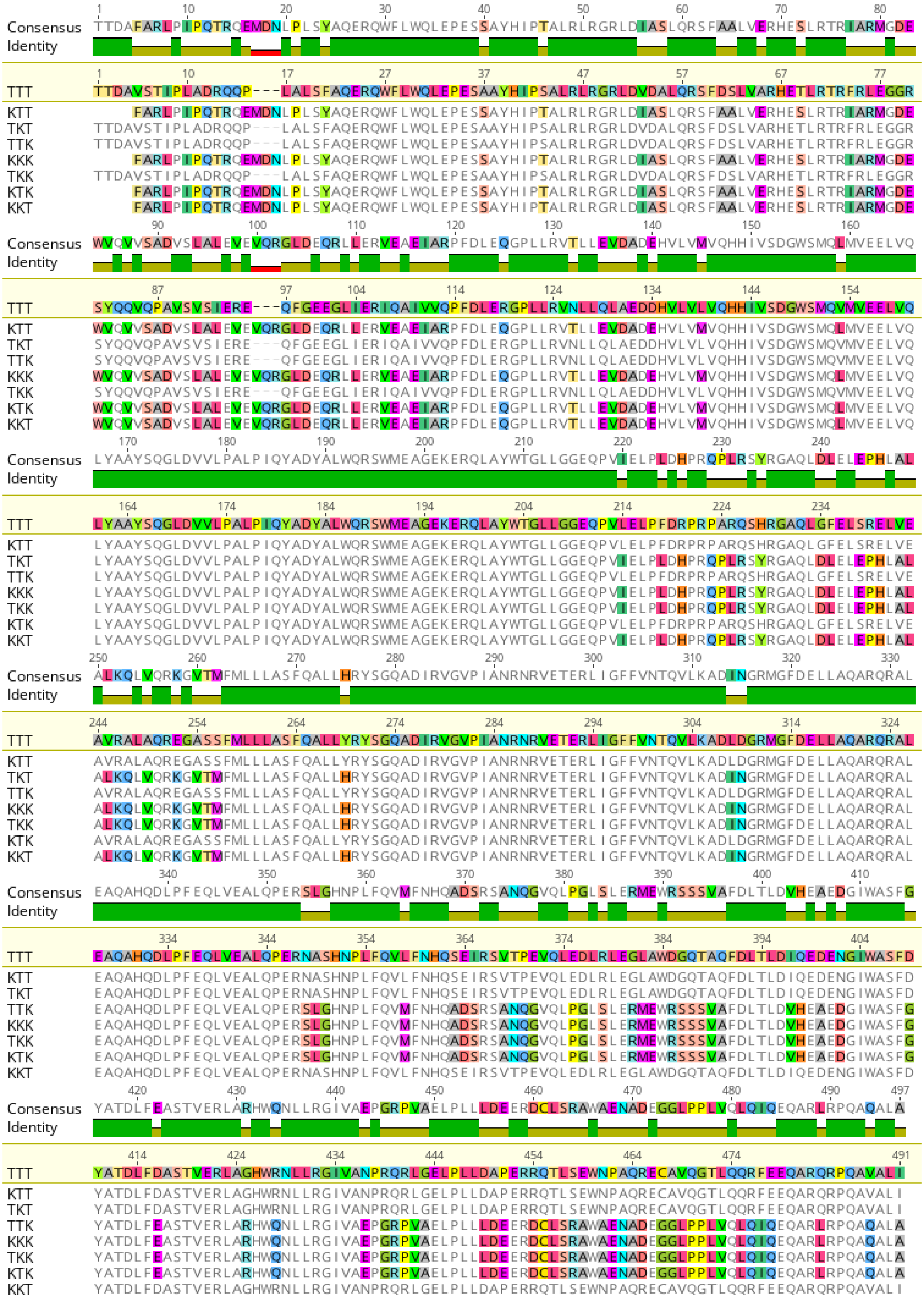

Supplementary Figure 3. Mass spectra for variants from semi-rational shuffling. Related to Figure 2c. Mass spectra from *P. aeruginosa* PAO1 *pvdD* deletion strains expressing *pvdD* constructs modified in module Pa11 by shuffling the three variable regions of the Pa8 and Pa11 C domains and placing them upstream to the (a) Thr-specific A domain from Pa11; or (b) Lys-specific A domain derived from module Pa8. Spectra are labelled according to the C domains in Figure 2C of the main text. Peaks corresponding to pyoverdine with a terminal Thr (1333.6 m/z) or Lys (1360.7 m/z) are highlighted. It is noted that the C domain labelled TTT produced trace levels of WT pyoverdine, detectable only by mass spectrometry. This is consistent with previous observations for a subset of cases where a non-cognate A domain was substituted into the Pa11 module.[1] We propose that trace amounts of wild type pyoverdine reflect a non-functional Pa11 module, and stem from iterative action of the Pa10 module allowing a second L-Thr to be incorporated with low efficiency. A total of n= 3 independent experiments were performed with consistent results; representative spectra are presented here.

a MS of shuffled C domains upstream to Thr-A domain

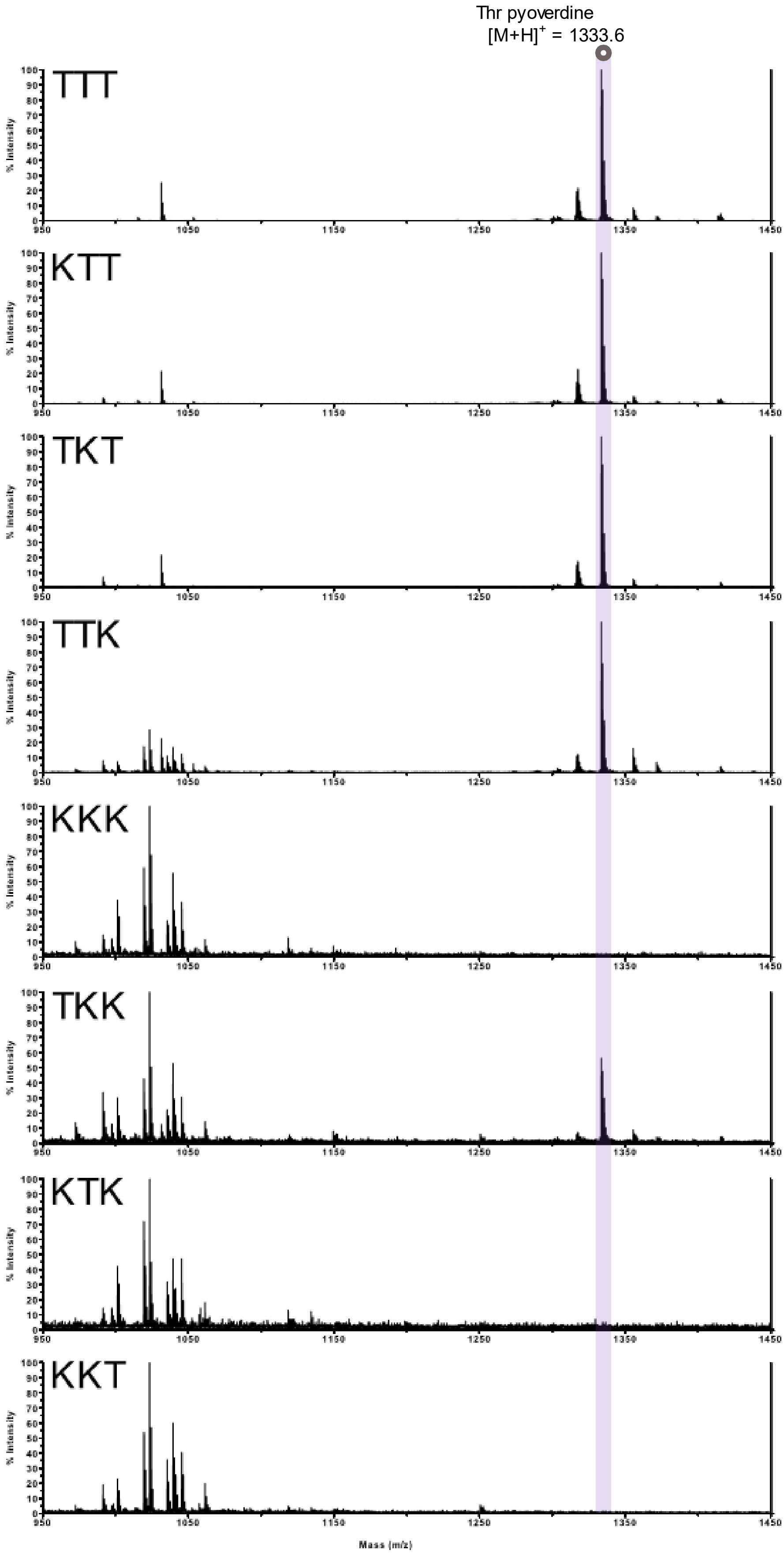

b MS of shuffled C domains upstream to Lys-A domain

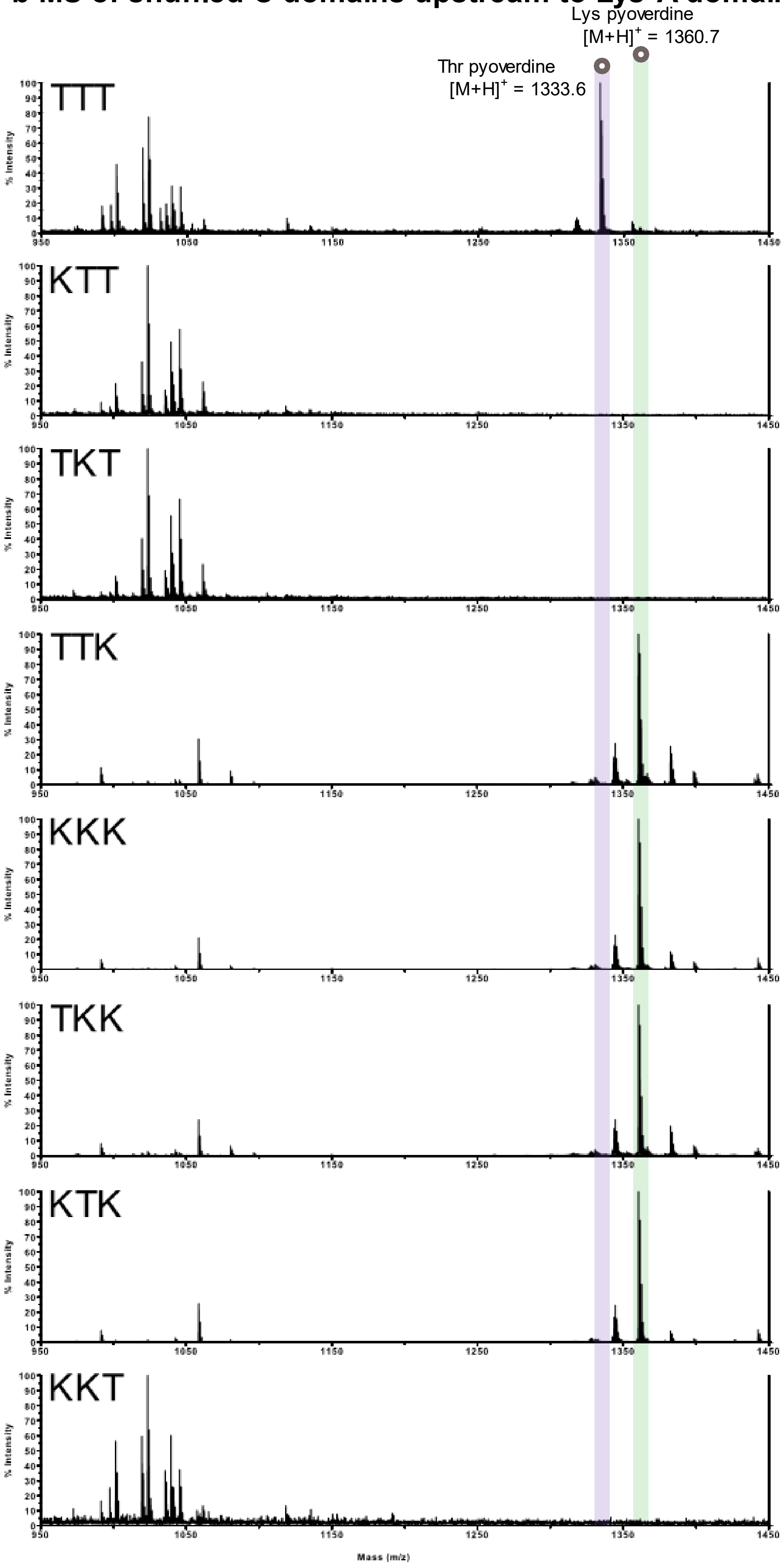

Supplementary Figure 4. Substitutions introduced into Pa11 during targeted mutagenesis of the third variable C domain region. Related to Figure 2e. Alignment of each variant generated by targeted mutagenesis against the equivalent region from Pa8 (provided for reference). Image created using Geneious version 8.1 (Biomatters. Available from http://www.geneious.com).

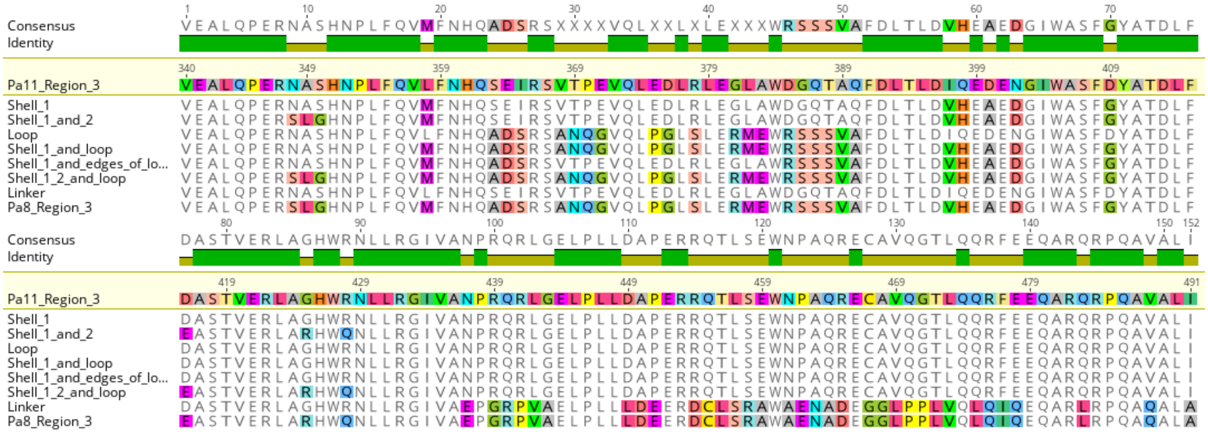

Supplementary Figure 5. Mass spectra for variants generated by mutating region 3 of the C domain. Related to Figure 2e. Mass spectra of pyoverdine species produced by *P. aeruginosa* PAO1 strains expressing *pvdD* constructs bearing a C domain mutated in region 3 and placed upstream to the (a) Thr-specific A domain of Pa11, or (b) Lys-specific A domain from Pa8. Spectra are labelled according to the C domain order provided in Figure 2e of the main text. Peaks corresponding to pyoverdine with a terminal Thr (1333.6 m/z) or Lys (1360.7 m/z) are highlighted. Similar to Supplementary Figure 3, trace amounts of wildtype pyoverdine were detected when low-yielding C domains were upstream of the Pa8 A domain. A total of n= 3 independent experiments were performed with consistent results; representative spectra are presented here.

a MS of region 3 mutations upstream to Thr-A domain

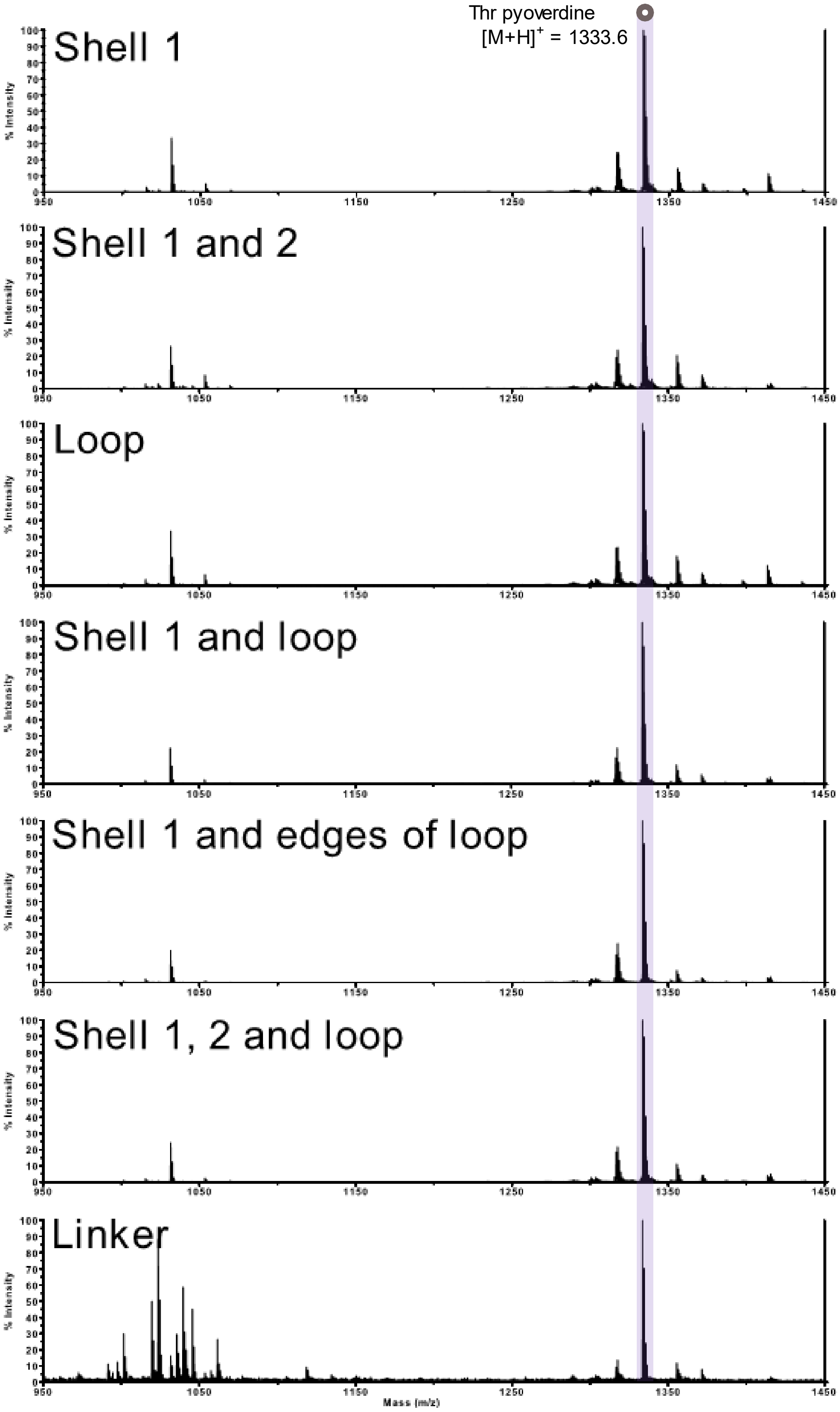

b MS of region 3 mutations upstream to Lys-A domain

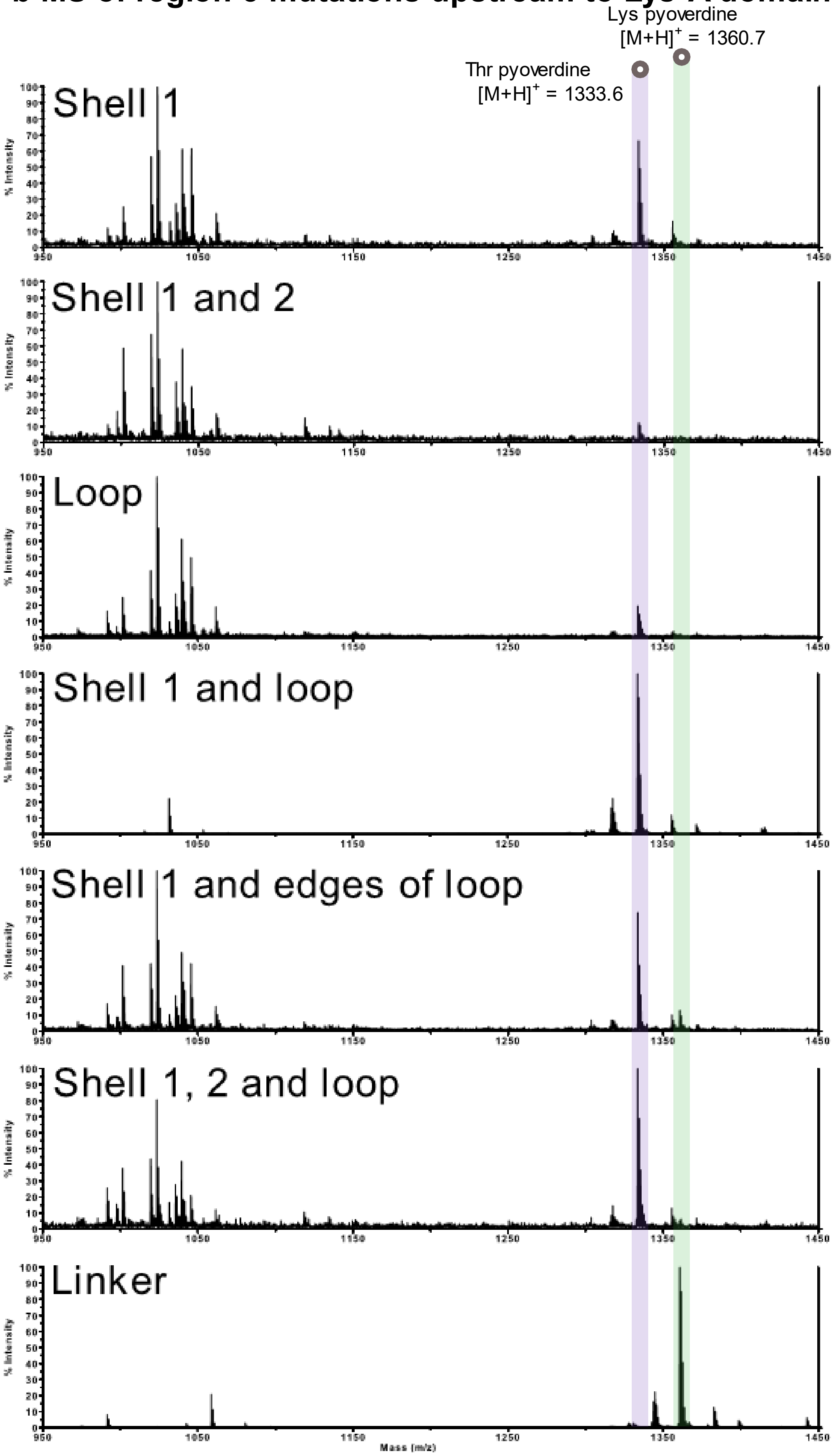

Supplementary Figure 6. Alignment of previously successful C-A domain substitutions with the C-A domains from Pa11. Related to Figure 3a. Each C-A domain pairing is labelled according to the module from which it was derived and its substrate specificity. The upstream recombination point used for linker + A domain substitution is labelled R1 (A436 in Pa11_CA_Thr). The C-A linker highlighted in red is defined as the sequence located between the terminal helix of the C domain and first helix of the A domain. Image created using Geneious version 8.1 (Biomatters. Available from http://www.geneious.com).

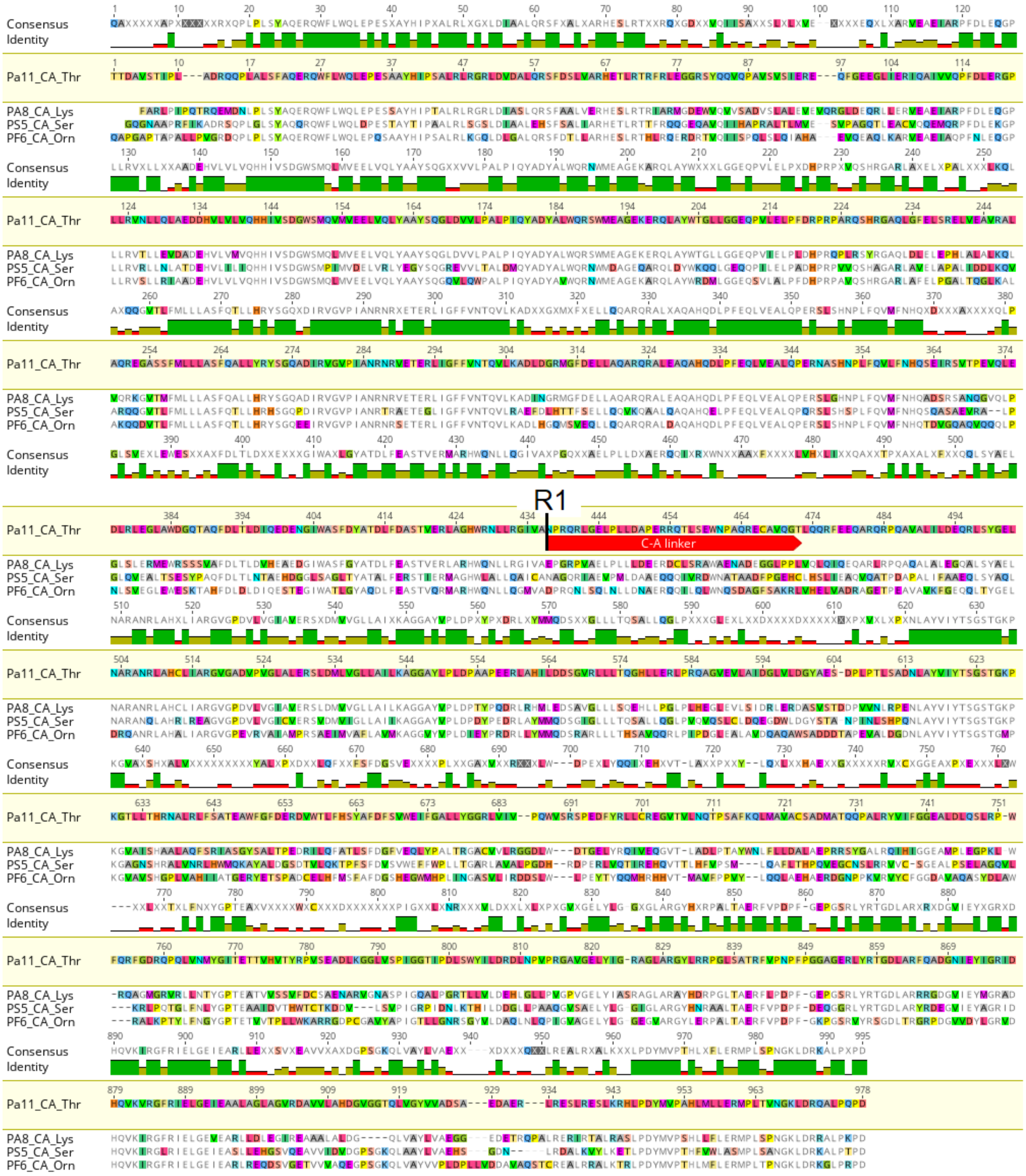

Supplementary Figure 7. Mass spectra for C-A and linker + A domain variants. Related to Figure 3a. Mass spectra of pyoverdine species produced by *P. aeruginosa* PAO1 strains expressing *pvdD* constructs bearing C-A domain or linker + A domain substitutions. Peaks corresponding to pyoverdine containing a terminal Lys (1360.7 m/z), Ser (1319.6 m/z) and N^5-formyl-N^5-hydroxyornithine (fhOrn; 1390.6 m/z) are highlighted. A total of n= 3 independent experiments were performed with consistent results; representative spectra are presented here.

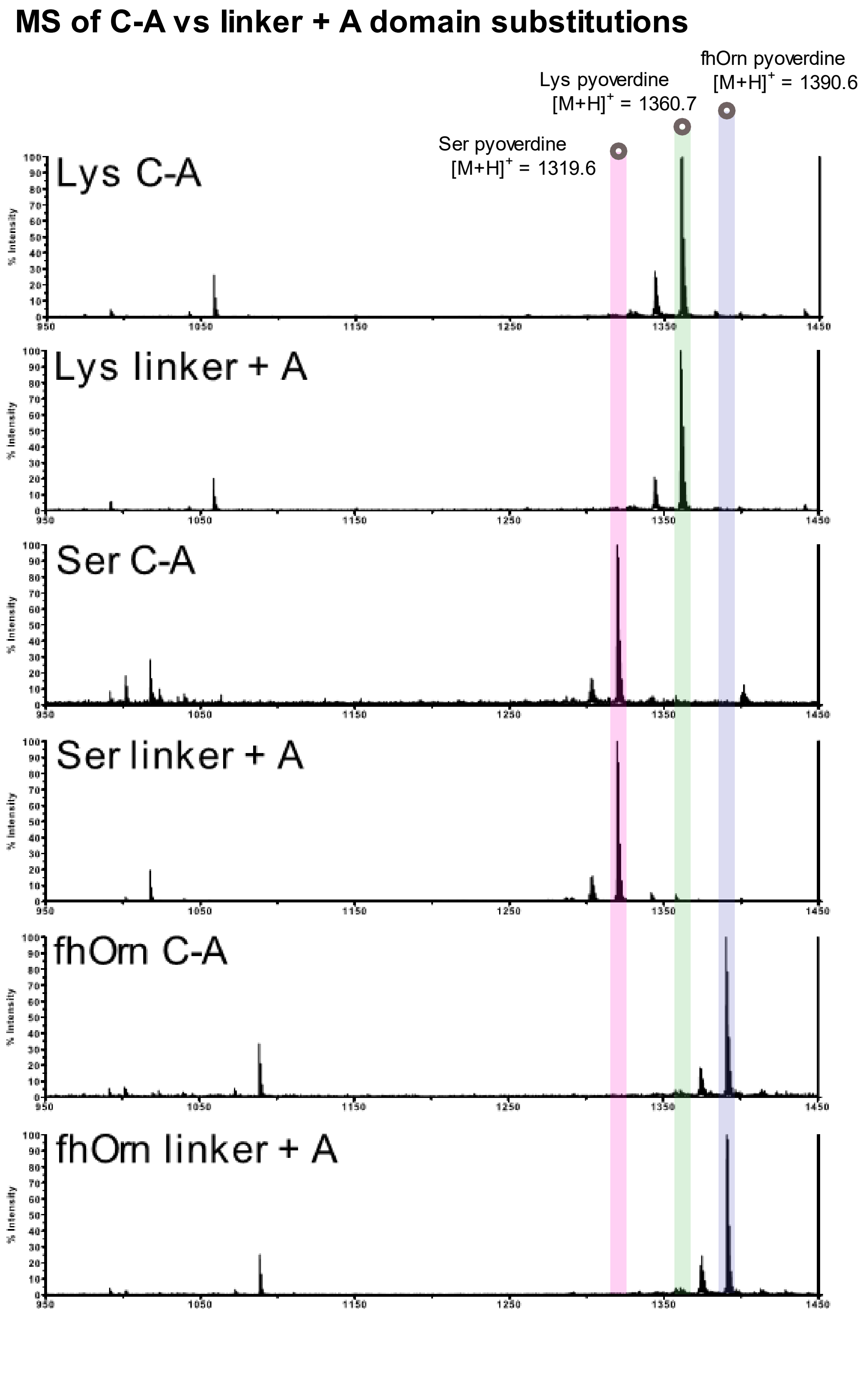

Supplementary Figure 8. Amino acid alignment highlighting differences between the C-A-T domains of module Pa11 and the C-A-T domains from modules used as a source for linker plus A domain substitution. Related to Figure 3b. The recombination points used for A domain substitutions are labelled R1 (N424 in Pa11_CAT) and R2 (D965 in Pa11_CAT). Image created using Geneious version 8.1 (Biomatters. Available from http://www.geneious.com).

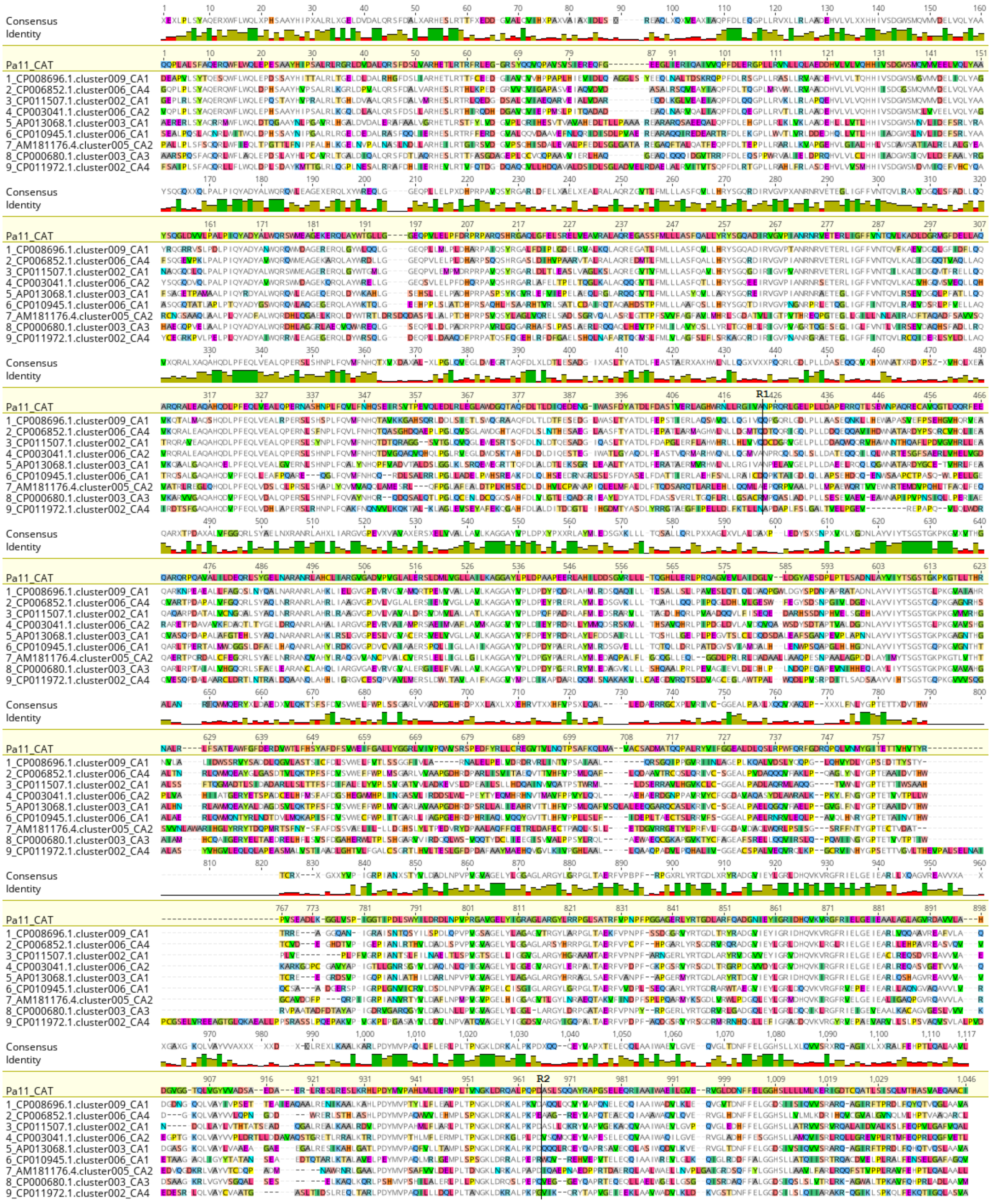

Supplementary Figure 9. Mass spectra for additional linker + A domain variants. Related to Figure 3b. Mass spectra of pyoverdine species produced by *P. aeruginosa* PAO1 strains expressing *pvdD* constructs bearing linker + A domain substitutions. Peaks corresponding to pyoverdine with a terminal Ala (1303.6 m/z), Ser (1319.6 m/z), N^5-formyl-N^5-hydroxyornithine (fhOrn; 1390.6 m/z) and Glu (1361.6 m/z). A total of n= 3 independent experiments were performed with consistent results; representative spectra are presented here.

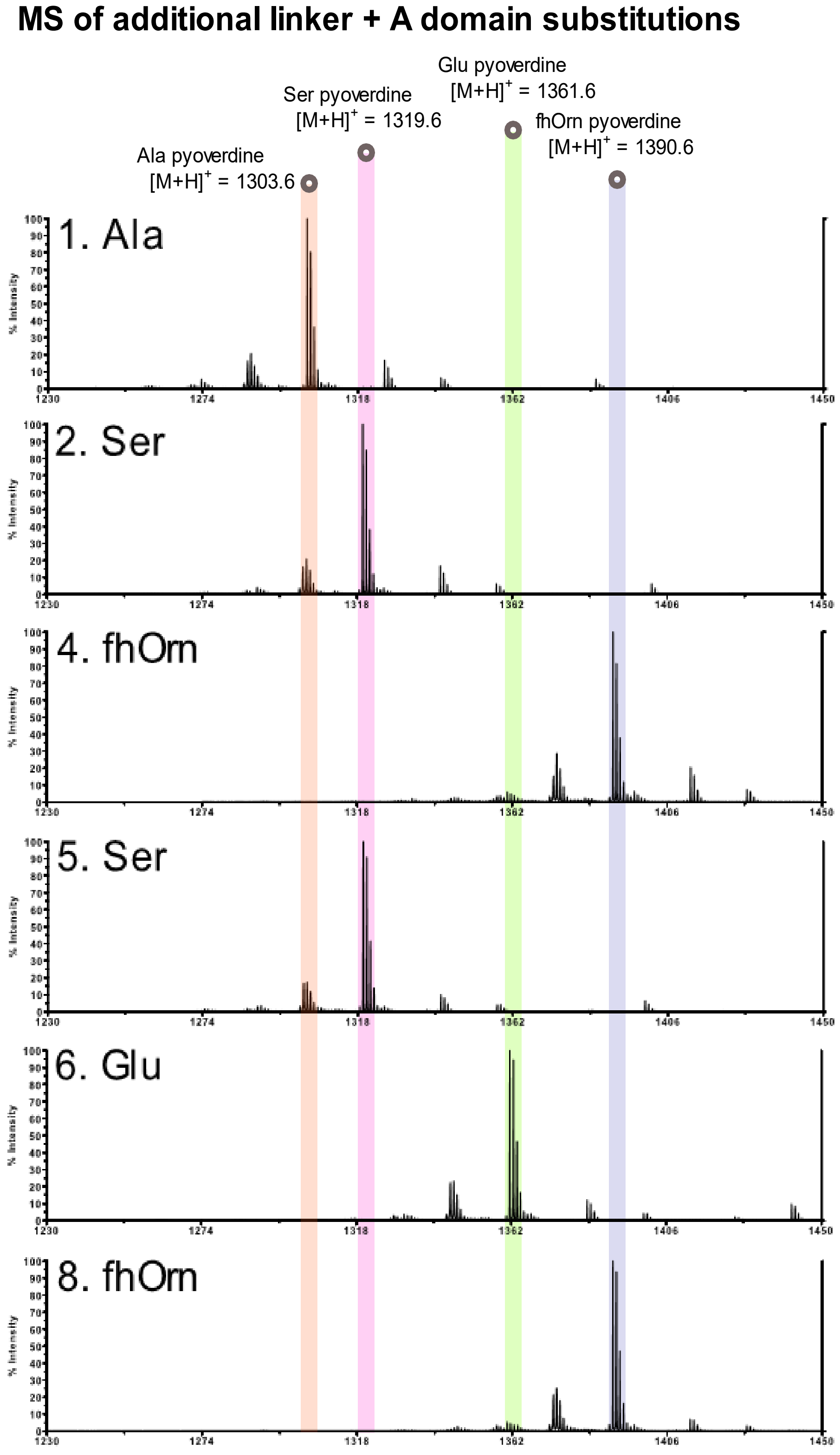

Supplementary Figure 10. Evidence for independent evolution of A domains and C domains in pyoverdine NRPS pathways. Related to Figure 4. (a) Maximum likelihood phylogenetic tree for the A domains from four pyoverdine pathways. Domains are labelled according to the names in Panel d and coloured according to the substrate specificity of the corresponding A domain. Letters A to J indicate modules having the same substrate specificity within the same pathway. (b) Maximum likelihood phylogenetic tree for the C domains from the same pathways presented in Panel a. (c) Key showing the colouring used to indicate substrate specificity in panels a and b. (d) Pyoverdine NRPS modules that were used as a source of sequences for phylogenetic analysis. Modules are from the pyoverdine NRPS pathways from i) *P. aeruginosa* PAO1, ii) *P. syringae pv. phaseolicola* 1448A, iii) *P. putida* KT2440 and iv) *P. fluorescens* SBW25. The substrate specificity and working name of each module is located below its position in the NRPS schematic. Modules within the same pathway that exhibit the same substrate specificity are linked by curved lines labelled A to J. Modules containing an $^{L}C_{L}$-domain are coloured red and those containing a $^{D}C_{L}$-domain are coloured blue.

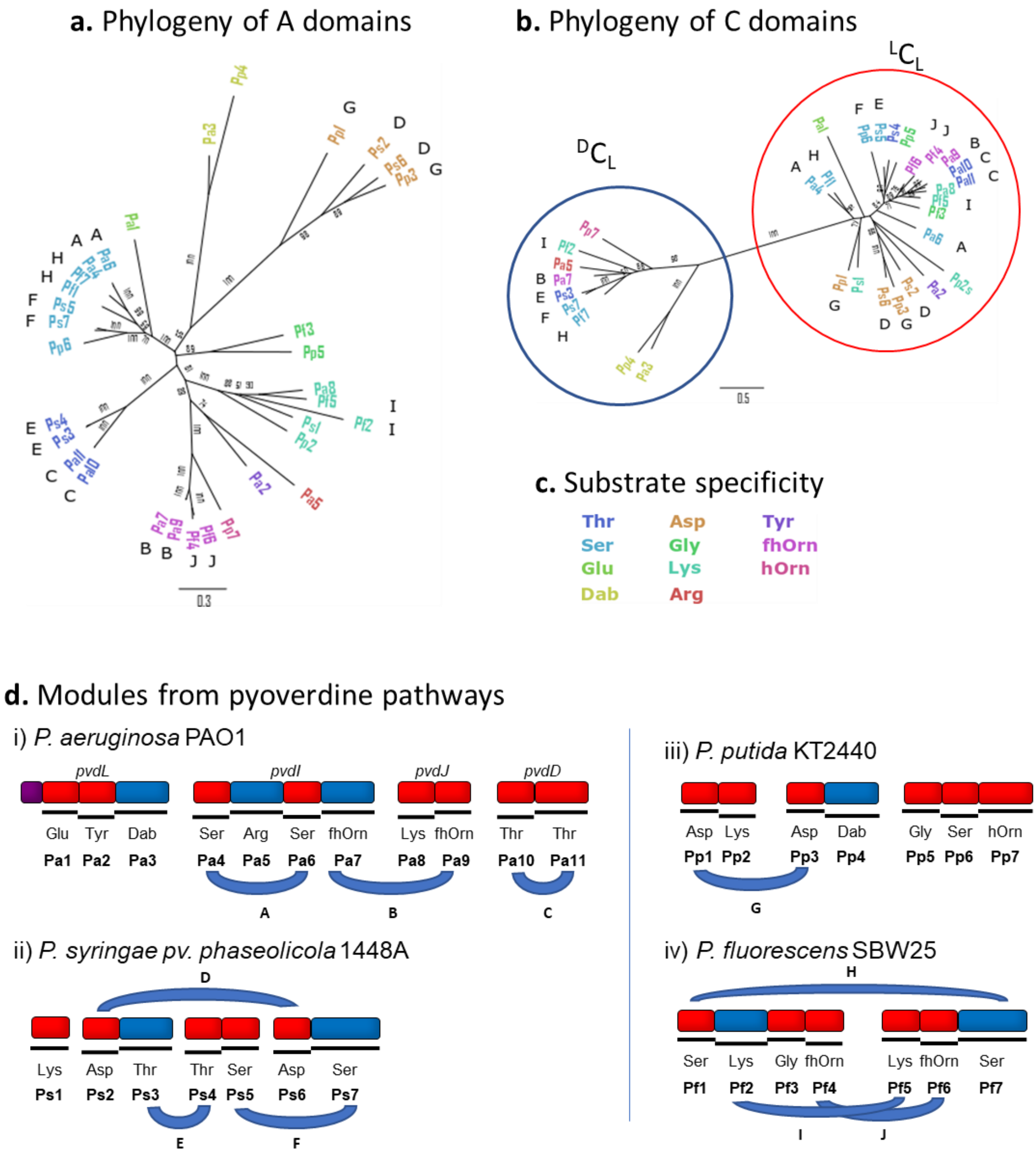

Supplementary Figure 11. Amino acid alignment highlighting changes to PvdD introduced by partial A domain substitutions. Related to Figure 4. Each partial A domain substitution construct is labelled according to the module from which it was derived and its substrate specificity, as per Supplementary Table 1. Image created using Geneious version 8.1 (Biomatters. Available from http://www.geneious.com).

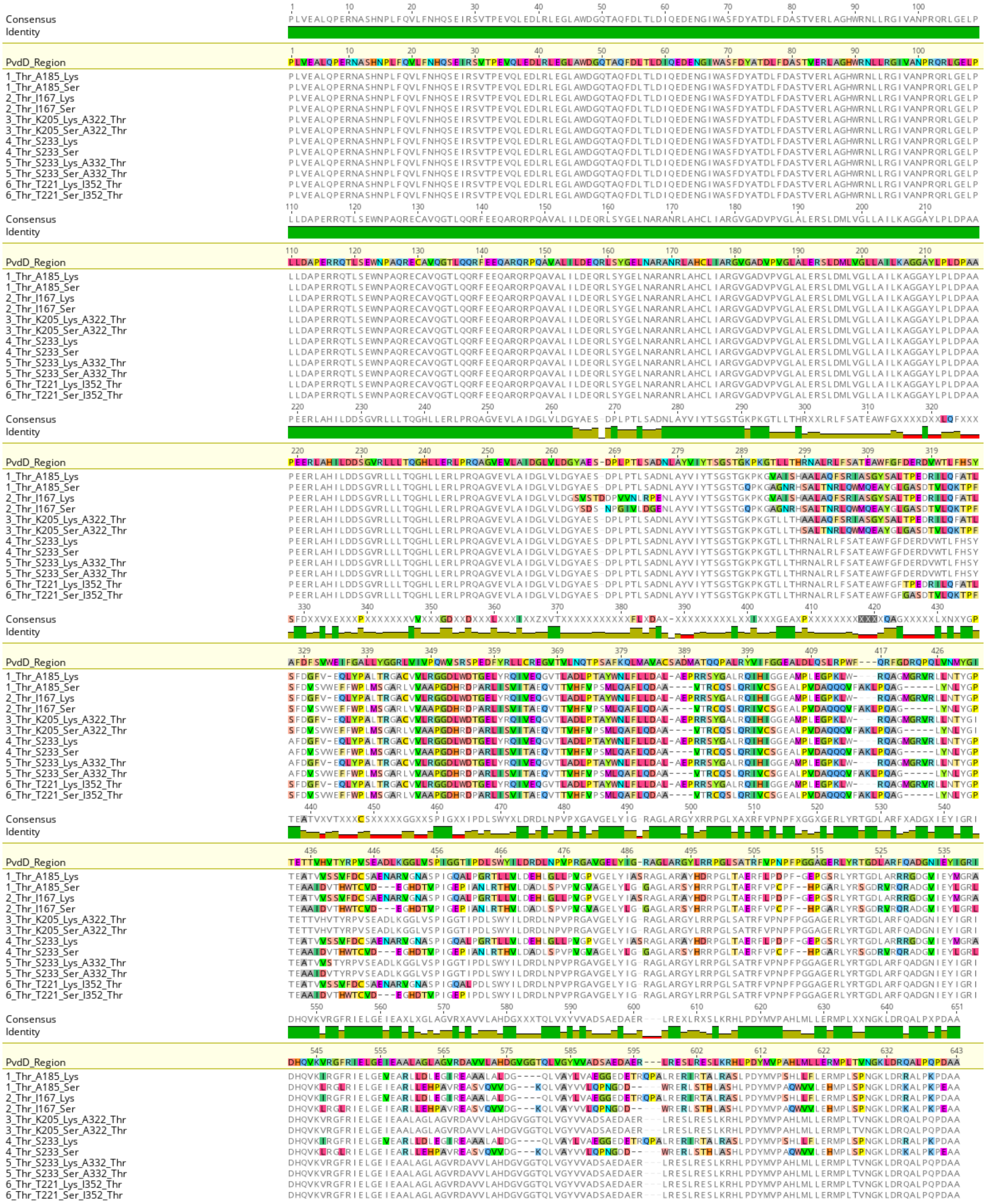

Supplementary Figure 12. Pyoverdine production yields for attempted partial A domain substitutions. Related to Figure 4d. (a) Schematic indicating the substituted regions for partial A domain substitutions in comparison to the SCHEMA profile from Figure 4d. (b) Pyoverdine production by *P. aeruginosa pvdD* deletion strains transformed by partial A domain substitution constructs. Samples are numbered as per Supplementary Figure 11. Pyoverdine levels were measured by optical density at 400 nm. A total of n= 3 independent experiments were performed and data are presented as mean values +/- SD. Source data are provided as a Source Data file.

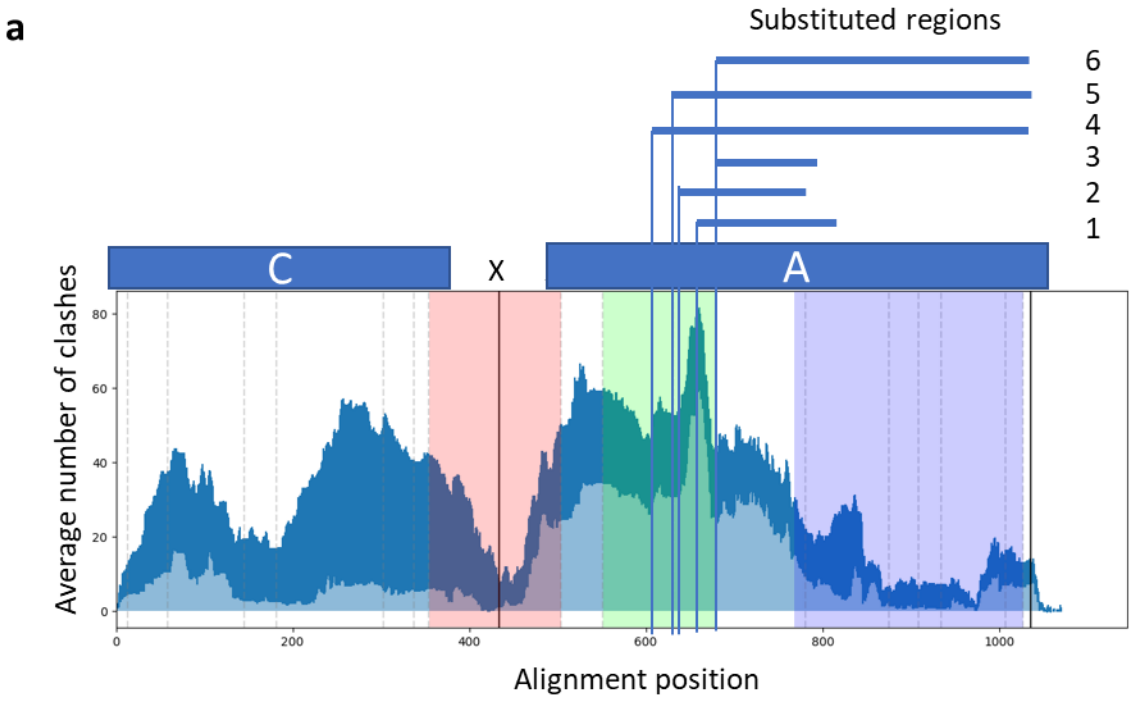

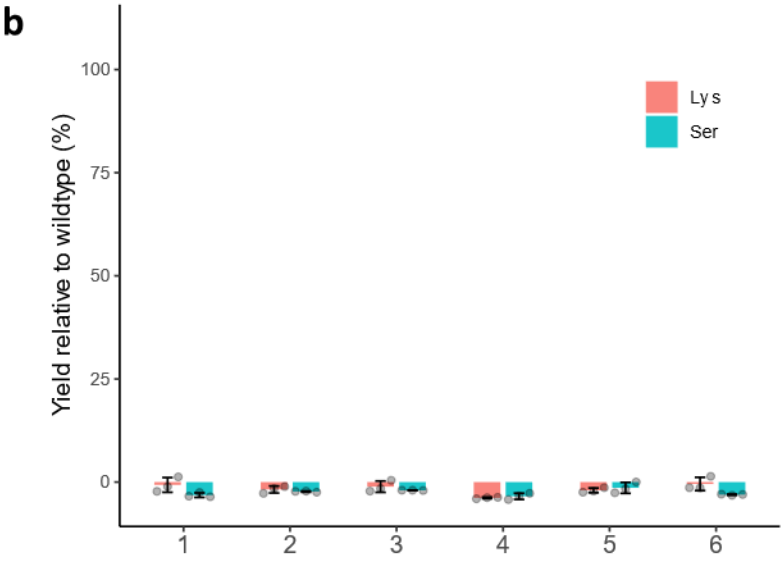

Supplementary Figure 13: Alignment of C-A-T domains used for substitutions in the PheATE-ProCAT system. Related to Figure 5. Amino acid alignment highlighting the differences between the C-A-T domains of ProCAT and the C-A-T domains from the Leu-specifying modules used for linker + A domain substitutions. The recombination points used for A domain substitutions are labelled R1 (N424 in TycB1_Pro) and R2 (E952 in TycB1_Pro). Image created using Geneious version 8.1 (Biomatters. Available from http://www.geneious.com).

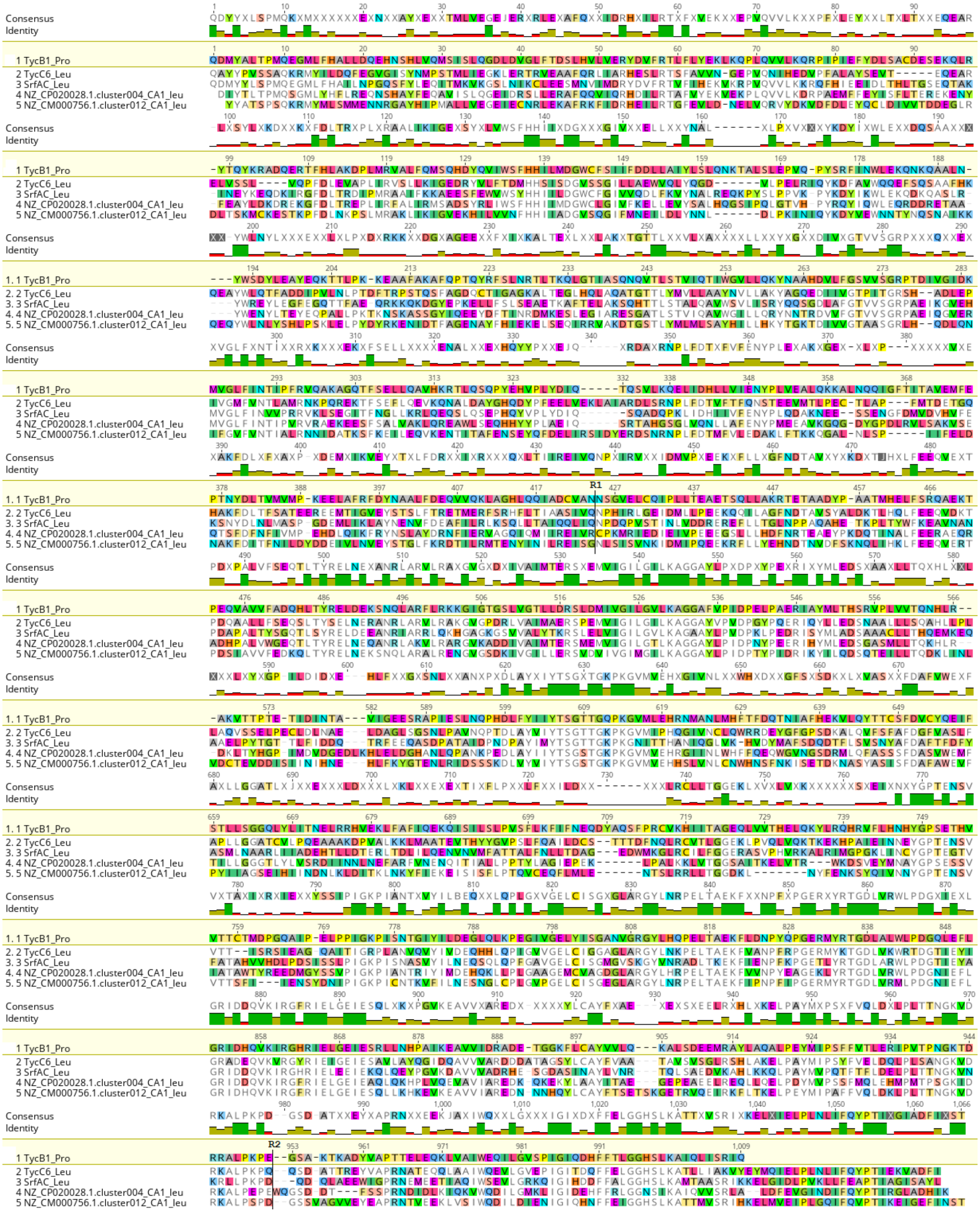

Supplementary Figure 14. Mass spectrometry traces for A domain substitution strains using the PheATE/ProCATTe system. Related to Figure 5. Total ion count and the EICs for Phe-Pro DKP ($[M+H]^+$ = 245.13) and Phe-Leu ($[M+H]^+$ = 279.17) are shown. Traces are labelled 1 to 5 as per Figure 5 in the main text. A total of n= 3 independent experiments were performed with consistent results; representative spectra are presented here.

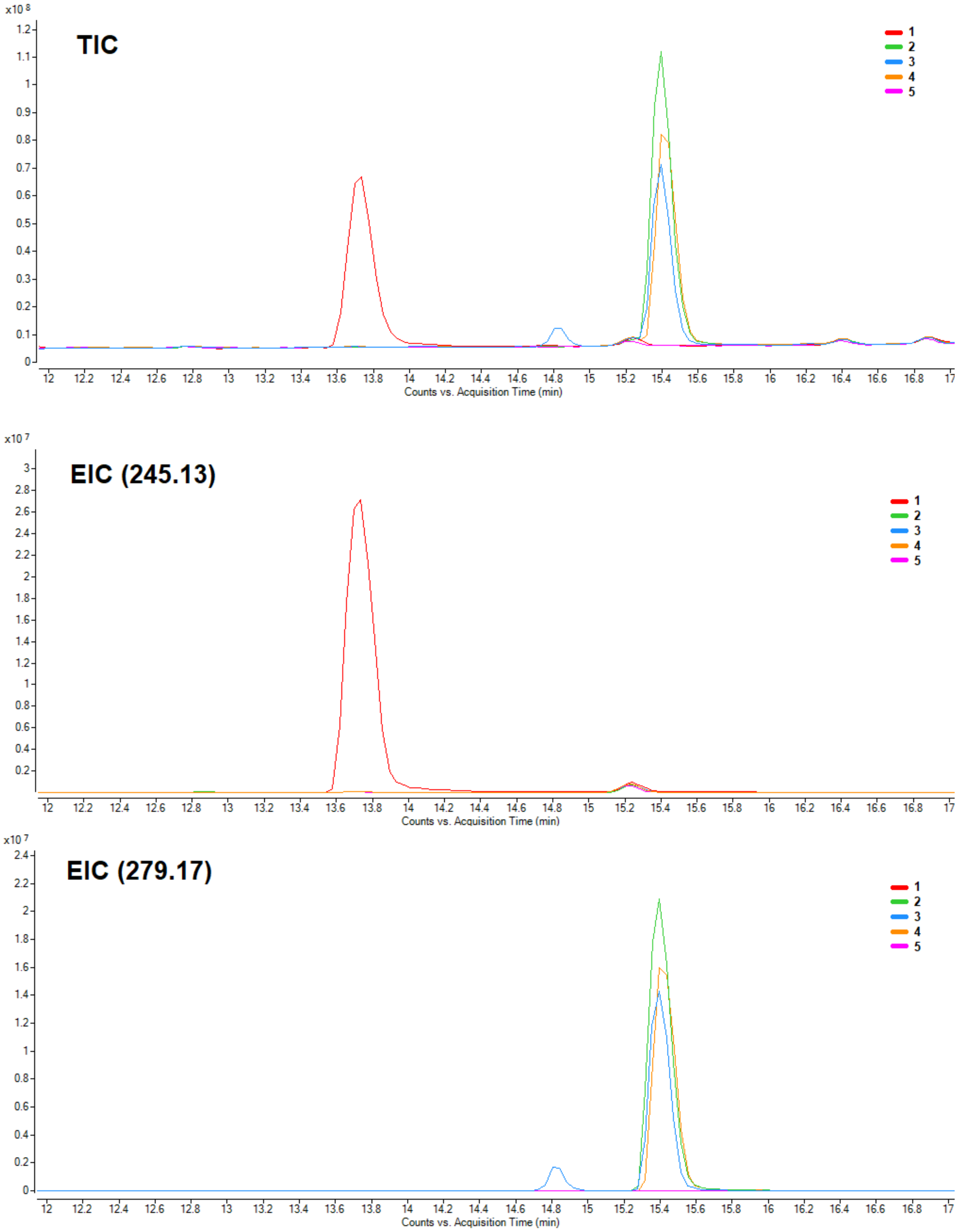

Supplementary Table 1. Substrate specificity predictions for each A domain substituted into PvdD. Additional information includes the name of each cluster, and amino acid identity shared between the C and A domains of each module and the corresponding domains from module Pa11-Thr.

NRPS Predictor2 SVM	Stachelhaus code	Minowa	Consensus	Cluster name*	Genbank accession and module position	C domain % identity	A domain % identity
ala	ala	ala	ala	1.CP008696.1.cluster009_CA1	WP_038633919 module 1	66.57	51.32
ser	ser	ser	ser	2.CP006852.1.cluster006_CA4	WP_081730346 module 4	72.24	50.54
gly	gly	gly	gly	3.CP011507.1.cluster002_CA1	WP_082224324 module 1	71.94	47.58
hydrophilic	orn	orn	orn	4.CP003041.1.cluster006_CA2	WP_168228785 module 2	73.43	47.01
ser	ser	ser	ser	5.AP013068.1.cluster003_CA1	WP_016492207 module 1	58.51	50.64
glu	glu	ser	glu	6.CP010945.1.cluster006_CA1	WP_017339971 module 1	53.61	48.39
asp, asn, glu, gln, aad	asp	orn	nrp	7.AM181176.4.cluster005_CA2	WP_012724386 module 2	42.99	43.98
hydrophilic	trp	orn	nrp	8.CP000680.1.cluster003_CA3	WP_012019058 module 4	53.13	47.39
asp	asn	asp	asp	9.CP011972.1.cluster002_CA4	WP_096059462 module 1	53.73	39.48

*Domains were named according to the cluster name from the antiSMASH database,[2] and a number based on the order the CA-domains appeared in the GBK file.

**C domains were trimmed to the C1 and C7 motifs, and A domains were trimmed to the A1 and A10 motifs inclusive. Domains were aligned using MUSCLE,[3] and the resulting alignment used to calculate percent identity to the corresponding sequence from module Pa11.

Supplementary Table 2. Substrate specificity predictions for each A domain substituted into ProCATTe. Additional information includes the name of each cluster, and amino acid identity shared between the C and A domains of each module and the corresponding domains from ProCATTe.

Specificity predictions using Stachelhaus code	Cluster name*	Genbank accession number of protein and module position	C domain % identity	A domain % identity
Leu	2.TycC6_Leu	O30409 module 6	22.98	43.11
Leu	3.SrfC_Leu	WP_062826629 module 1	44.08	40.35
Leu	4.NZ_CP020028.1.cluster004_Leu_CA1	WP_094154864 module 2	43.07	47.02
Leu	5.NZ_CM000756.1.cluster012_Leu_CA1	WP_000023653 module 3	21.82	47.64

*Domains were named according to the cluster name from the antiSMASH database.[2]

**C-domains were trimmed to the C1 and C7 motifs, and A domains were trimmed to the A1 and A10 motifs inclusive. Domains were aligned using MUSCLE,[3] and the resulting alignment used to calculate percent identity to the corresponding sequence from ProCAT.

Supplementary Table 3. The numbers of $^{L}C_{L}$-A-T sequences employed prior to and during each step of processing.

	Pseudomonas	*Streptomyces*	*Bacillus*
Sequences	824	294	1493
Unique sequences	758	253	1067
Clusters at 95%	453	222	371
After alignment	437	213	370

References

1 Calcott, M. J., Owen, J. G., Lamont, I. L. & Ackerley, D. F. Biosynthesis of novel pyoverdines by domain substitution in a nonribosomal peptide synthetase of *Pseudomonas aeruginosa*. *Appl Environ Microb* **80**, 5723-5731, doi:10.1128/Aem.01453-14 (2014).

2 Blin, K., Medema, M. H., Kottmann, R., Lee, S. Y. & Weber, T. The antiSMASH database, a comprehensive database of microbial secondary metabolite biosynthetic gene clusters. *Nucleic Acids Res* **45**, D555-D559, doi:10.1093/nar/gkw960 (2017).

3 Edgar, R. C. MUSCLE: multiple sequence alignment with high accuracy and high throughput. *Nucleic Acids Res* **32**, 1792-1797, doi:10.1093/nar/gkh340 (2004).
