# Supplementary material for: Efficient rational modification of non-ribosomal peptides by adenylation domain substitution
Source: Nat Commun. 2020 Sep 11;11:4554. doi: 10.1038/s41467-020-18365-0 (PMC7486941; doi:10.1038/s41467-020-18365-0)
Supplement: Supplementary file 3 — Descriptions of Additional Supplementary Files [file 41467_2020_18365_MOESM3_ESM.pdf]

Descriptions of Additional Supplementary Files

Supplementary Data 1

Description: Excel file of Plasmids, Primers and Gene Sequences used in this study.
